# NK Cell-Based Immunotherapy in Cancer Metastasis

**DOI:** 10.3390/cancers11010029

**Published:** 2018-12-28

**Authors:** Seila Lorenzo-Herrero, Alejandro López-Soto, Christian Sordo-Bahamonde, Ana P Gonzalez-Rodriguez, Massimo Vitale, Segundo Gonzalez

**Affiliations:** 1 Department of Functional Biology, Immunology, University of Oviedo, 33006 Oviedo, Spain; seilalorenzoherrero@gmail.com (S.L.-H.); lopezsoa@gmail.com (A.L.-S.); christiansbl87@gmail.com (C.S.-B.); 2 Instituto Universitario de Oncología del Principado de Asturias, IUOPA, 33006 Oviedo, Spain; anapilargonzalez@gmail.com; 3 Instituto de Investigación Biosanitaria del Principado de Asturias (IISPA), 33011 Oviedo, Spain; 4 Department of Hematology, Hospital Universitario Central de Asturias (HUCA), 33011 Oviedo, Spain; 5 UOC Immunologia, Ospedale Policlinico San Martino Genova, 16132 Genoa, Italy; massimo.vitale@hsanmartino.it

**Keywords:** cancer, metastasis, epithelial-to-mesenchymal transition (EMT), therapy, natural killer cell, checkpoint, chimeric antigen receptor (CAR), adoptive transfer, cytokines, antibodies

## Abstract

Metastasis represents the leading cause of cancer-related death mainly owing to the limited efficacy of current anticancer therapies on advanced malignancies. Although immunotherapy is rendering promising results in the treatment of cancer, many adverse events and factors hampering therapeutic efficacy, especially in solid tumors and metastases, still need to be solved. Moreover, immunotherapeutic strategies have mainly focused on modulating the activity of T cells, while Natural Killer (NK) cells have only recently been taken into consideration. NK cells represent an attractive target for cancer immunotherapy owing to their innate capacity to eliminate malignant tumors in a non-Major Histocompatibility Complex (MHC) and non-tumor antigen-restricted manner. In this review, we analyze the mechanisms and efficacy of NK cells in the control of metastasis and we detail the immunosubversive strategies developed by metastatic cells to evade NK cell-mediated immunosurveillance. We also share current and cutting-edge clinical approaches aimed at unleashing the full anti-metastatic potential of NK cells, including the adoptive transfer of NK cells, boosting of NK cell activity, redirecting NK cell activity against metastatic cells and the release of evasion mechanisms dampening NK cell immunosurveillance.

## 1. Introduction

Natural Killer (NK) cells are cytotoxic immune cells with an innate capacity for eliminating transformed cells in a non-major histocompatibility complex (MHC) and non-tumor antigen-restricted manner [1]. The activation of NK cells depends on a balance of signals provided by inhibitory and activating receptors that detect changes in the patterns of expression of their ligands on the surface of tumor cells. Inhibitory NK cell receptors recognize self-proteins and transmit inhibitory signals that maintain tolerance to normal cells [1,2]. Killer cell immunoglobulin-like receptors (KIRs) and the heterodimer CD94-Natural Killer Group 2A (NKG2A) are inhibitory receptors that recognize self-MHC class I molecules, whereas other inhibitory receptors, such as T cell immunoreceptor with Ig and ITIM domains (TIGIT) receptor, bind to other self-molecules [2,3,4]. Transformed cells frequently downregulate MHC class I molecules, thereby avoiding recognition by CD8+ cytotoxic T cells, but concomitantly inducing the activation of NK cells by missing self-recognition. Activating receptors, including, but not limited to, killer cell lectin-like receptor K1 (KLRK1—best known as NKG2D), DNAX accessory molecule-1 (CD226—best known as DNAM-1) and the natural cytotoxicity receptors NKp46, NKp44, and NKp30, recognize stress-inducible ligands on tumor cells that are scarcely expressed in their normal counterparts [2,3,4,5]. Natural killer group 2D (NKG2D) is a particularly relevant activating receptor, which recognizes a group of stress-inducible molecules termed MHC class I polypeptide-related sequence A and B (MICA and MICB) and UL16 binding protein molecules (ULBP1-6), which are restrictedly expressed on stressed and transformed cells [6,7]. Thus, by this complex pattern of receptors, NK cells may kill a broad range of cancer cells. Indeed, the engagement of activating receptors by tumor-expressed ligands, along with a lack of co-engagement of an appropriate number of inhibitory receptors, results in the exocytosis of cytotoxic granules containing perforin and granzymes that induce apoptotic cell death of the target cells. Additionally, NK cells can eliminate target cells through Fas ligand and tumor necrosis factor (TNF)-related apoptosis-inducing signals [2,3]. Finally, NK cells may also kill tumor cells bound by specific IgG antibodies through FcγRIII receptors (also named as CD16s), a process known as antibody-dependent cellular cytotoxicity (ADCC). The latter is a relevant process underlying the therapeutic activity of certain monoclonal antibodies [8]. NK cells also regulate the innate and adaptive immune response through the secretion of cytokines with potent antitumor activity, such as interferon-gamma (IFN-γ). 

## 2. The Metastatic Cascade

The metastatic cascade involves tumor cell detachment from their neighbors, local invasion of surrounding tissues—as either a singular cells or groups of tumor cells—and the tumors entrance into the nearby pre-existing or neo-formed vasculature. In this scenario, typical features of such aggressive tumor cells are the partial loss of epithelial markers and the acquisition of a mesenchymal-like phenotype typically associated with migratory and invasive properties, which happens during the so-called epithelial-to-mesenchymal transition (EMT) (see below) [9]. Upon intravasation into the vascular bed, single or circulating tumor cell (CTC) clusters travel throughout the circulatory system until they lodge at secondary sites and extravasate, via transendothelial migration, through capillary walls. Once at the target site, disseminated tumor cells (DTCs) can survive for a short period or for decades as dormant and indolent entities before micrometastasis proliferation is triggered, giving rise to overt macrometastasis and organ colonization [10].

Metastasis formation is an extremely selective process wherein a progressive decimation of the tumor population occurs. The metastatic cascade may be envisioned as a sequence of selective microenvironments demanding specific malignant attributes. Among these tumor-extrinsic factors, the host’s anti-tumor immune response is a major hindrance that cancer cells must avoid to successfully seed distant metastases. In fact, owing to cancer immunosurveillance, a myriad of immune components can be detected at every step of the metastatic process as a forced companion of tumor cells, an immune contexture that constitutes a strong prognostic factor of the outcome of the disease [11]. This pairing results in a complicated bidirectional relationship whereby the immune system shapes the immunogenicity of the developing tumor by operating both tumor-promoting and tumor-obstructive mechanisms throughout the metastatic process. 

## 3. NK Cells and the Immunosurveillance of Metastasis

Accumulating data evidence a central role of NK cells in the control of metastasis in preclinical models [12]. Nevertheless, NK cells show a poor capacity for infiltrating and eliminating large solid tumors in humans [13,14,15,16,17]. Indeed, NK cells are scarcely present in the tumor bed of colorectal cancers, despite the high levels of local chemokines and the normal numbers of NK cells observed in adjacent mucosa [18]. Moreover, tumor-infiltrating NK cells are frequently enriched by poorly cytotoxic CD56^bright^ or poorly functional CD56^dim^ NK cells with a decreased capacity for eliminating tumor cells [19,20,21,22]. Furthermore, NK cells are preferentially located in the stroma, rather than in direct contact with tumor cells in colorectal carcinomas, melanomas and Gastro-Intestinal Stromal Tumors (GISTs) [17,23]. It has recently been reported that tumor hyperploidy induced NK cell antitumor activity occurs mainly through the activation of NKG2D and DNAM-1 receptors [24] and that the level of aneuploidy negatively correlated with the NK cell infiltration of solid tumors, providing rational support for these observations [25]. The abovementioned observations notwithstanding, NK cells are sometimes successful in infiltrating primary tumors. The level of NK cell infiltration was associated with better prognoses of metastatic colorectal carcinomas and metastatic renal cell carcinomas [26,27]. There is a correlation between the density of infiltrating NK cells, metastasis, and the patient’s prognosis in terms of esophageal cancer, gastric carcinomas, and prostate cancer [28,29,30]. Furthermore, patients with prostate carcinomas with high NKp46 and NKp30 expression on intra-tumoral NK cells showed a high tendency toward no progression 1 year after surgery [22,31]. The levels of NKp46+ cell infiltration inversely correlated with the presence of metastases upon the diagnosis of gastrointestinal sarcoma tumors [32]. 

Along the same thread, the immune evasion mechanisms developed by tumor cells, including the downregulation of NK cell activating receptors or the shedding of NK cell activating ligands (NKARLs) as soluble proteins, are associated with poor prognosis and metastasis of several types of cancer (see below). Similarly, dendritic cell (DC)-induced IFN-γ production by NK cells, as well as the expression of the NKp30 splice variant (immunosuppressive NKp30C variant vs. immunostimulatory NKp30A and B variants), was found to constitute an independent predictor of the long term survival of patients with advanced gastrointestinal stromal cancer treated with Imatinib Mesylate [33] and those with neuroblastoma treated with chemotherapy [34]. NK cells have also been involved in the control of metastasis after cancer surgery [35]. Major surgery induces significant immunosuppression, mainly associated with the profound suppression of NK cells affecting both cytotoxic activity and cytokine production, thereby increasing the risk of metastasis in preclinical models and patients with cancer [35]. 

NK cells are present in lymph nodes and blood and may participate in the immunosurveillance of disseminated cells in the metastatic cascade. In melanoma patients, NK cells with cytotoxic capacity are recruited or generated in tumor-draining lymph nodes suggesting a potential role in metastasis immunosurveillance, although their prognostic significance has not yet been established [21,36,37]. Furthermore, the majority of tumor cells that enter into circulation are eliminated by NK cells within the first 24 h [38]. Hence, the number of circulating NK cells correlated with the number of CTCs and metastasis in triple-negative breast cancer and pancreatic cancer patients [39,40]. Conversely, and consistent with the immunoediting process, the activity of circulating NK cells generally decreases as the cancer progresses, thereby increasing the risk of metastatic disease in prostate cancer pharyngeal, carcinoma and gastric cancer patients [31,41,42]. These data, along with supporting the prominent role of NK cells in the control of metastasis, display an opportunity for immunotherapeutic intervention in cancer patients.

## 4. EMT and NK Cells

EMT is one of the key steps of the metastatic cascade whereby carcinoma cells lose epithelial characteristics and acquire a mesenchymal phenotype, which endows tumor cells with invasiveness, motility and resistance to apoptosis [9]. In addition, activation of the EMT program impinges on the immunomodulatory properties and the immunogenicity of cancer cells [43,44]. For instance, we reported that the induction of EMT in a colorectal cancer cell line, by the forced expression of the master regulator of the EMT process Snail family transcriptional repressor 1 (SNAI1; best known as Snail), is associated with the increased immunogenicity of tumor cells in vitro [45]. Furthermore, immunohistochemistry analyses showed that in well-differentiated colorectal tumors preserving the epithelial architecture, MICA/B was confined to the luminal part of the epithelial layer, thereby impeding the interaction with NKG2D-expressing immune cells located in the basal part of the epithelial tissue [45]. Conversely, disruption of the epithelial phenotype during EMT was associated with the loss of the polarized expression of MICA/B, likely rendering tumor cells sensitive to immune elimination. In fact, a significant decrease of MICA/B expression and a marked increase of NKG2D+ tumor-infiltrating lymphocytes were observed in less-differentiated tumors with mesenchymal characteristics. These suggest that the EMT process may constitute a cancer immune checkpoint and that colorectal tumor cells must develop mechanisms to escape from NKG2D-mediated immune responses in order to progress through the metastatic process [45,46]. Along similar lines, prostate cancer cells were shown to strongly downregulate MHC class I expression during TGF-β/EGF-induced EMT, rendering tumor cells resistant to CD8 T cell-mediated 0cytotoxicity, but increasing their susceptibility to NK cells in a process independent of Snail transcription factor [47]. In lung cancer, TGF-β-induced EMT confers increased tumor cell susceptibility to NK cell killing activity through the induction of E-cadherin and cell adhesion molecule 1 (CADM1) expression [48]. Accordingly, transplantation of epithelial tumors expressing *neu* oncogene into syngeneic mice induced an immune-mediated rejection of cancer cells [49]. Consistent with cancer immunoediting, these mice subsequently relapsed with tumors enriched in *neu*-negative variant cancer cells with a mesenchymal phenotype. These data together suggest that the EMT transdifferentiation may be an immune checkpoint crucial to the control of metastasis by NK cells. 

NK cells may control the development of cancer, principally during the initial steps of malignant transformation, but, in a specific tumorigenic context and mainly in the last stages of tumor transformation, they may also favor tumor progression [23]. In line with this, Huergo-Zapico and colleagues recently showed the unexpected role of NK cells in the promotion of pro-metastatic features of melanoma cells through the triggering of the EMT process, thereby promoting a tumor phenotype switching from proliferative to invasive [50]. NK cells were found to increase tumor resistance to NK cell-mediated killing by inducing the expression of NK cell-inhibitory MHC class I molecules on the surface of melanoma cells. These changes were mostly dependent on NKp30 or NKG2D engagement and release of IFN-γ and TNF-α by NK cells. Worth noting was the expression of the inhibitory immune checkpoint programmed death ligand 1 (CD274—best known as PD-L1), induced by IFN-γ produced by activated immune cells, including NK cells, which constitutes a prominent mechanism of tumor “adaptive resistance” to immunosurveillance [51]. Interestingly, PD-L1 expression has been reported to be downregulated by the EMT-repressor microRNA-200 (miR-200) in Non-Small-Cell Lung Carcinoma (NSCLC) [52,53] and breast carcinoma cells [54], hence unveiling a link between inhibitory immune checkpoint expression and the acquisition of a mesenchymal phenotype in cancer. Accordingly, a number of studies demonstrate a correlation between PD-L1 expression and EMT score in several types of malignancies, such as lung cancer and breast carcinomas, suggesting that the group of patients in whom malignant progression is driven by EMT activators may respond to treatment with PD1/PD-L1 antagonists [53]. Overall, the EMT process may have crucial influence over the immunosurveillance of cancer mediated by NK cells, hence opening a potential new window for therapeutic intervention. 

## 5. Metastasis and Evasion of NK Cell Surveillance

Immune evasion is a hallmark of cancer and metastatic cells develop the most refined de facto immunosubversive mechanisms [55]. Thus, in patients with advanced cancers, tumor cells exhibit decreased expression of NKARLs. Consequently, metastatic cancer cells are more likely to escape from NK cell antitumor surveillance, thereby increasing the probability of malignant dissemination. A manifold program of suppressive mechanisms has been reported to reduce NKARL expression in cancer, including, but not limited to, the proteolytic shedding of soluble NKARLs as well as epigenetic changes involving histone deacetylation [56] or microRNA overexpression [57,58,59]. Shedding of soluble MICA depends on its interaction with the chaperon molecule protein disulfide isomerase family A member six (PDIA6—best known as ERp5) on the surface of tumor cells [60]. ERp5 forms a transitory disulphide bond with MICA, which induces a conformational change in its α3 domain. This allows the proteolytic cleavage of MICA by proteases, including ADAM10, ADAM17 and MMP14, which are overexpressed in cancer cells [61,62]. ERp5 that had been identified as a metastasis-promoting factor in a mouse model of breast cancer was highly detected in human samples of invasive breast cancer [63]. Further, membrane ERp5 was functionally associated with soluble MICA shedding in chronic lymphocytic leukemia patients [64] and enhanced levels of soluble MICA correlated with membrane ERp5 expression in myeloma and lymphoma cells [65,66]. 

It has been widely reported that low cell surface expression of MICA/B or elevated sera levels of soluble MICA and MICB correlate with metastasis in different types of cancer [67,68,69,70,71,72,73,74]. Elevated sera levels of soluble ULBP2 are an indicator of progression in melanoma patients [75]. Low expression of ULBP4 also favors metastasis in nasopharyngeal carcinomas [76]. By contrast, the tumor tissue expression levels of B7-H6, a ligand of the activating NCR receptor NKp30, correlated with the metastasis and cancer progression of ovarian cancer [77]. Meanwhile, the serum concentration of soluble B7-H6 correlated with the down-regulation of NKp30, bone marrow metastasis, and chemo-resistance in high-risk neuroblastoma patients [34]. NK cells also expressed inhibitory receptors, such as PD1, which participated in the suppression of NK cell activity in advanced and metastatic patients [78,79,80].

Immunosuppressive factors also played a crucial role in the evasion of NK cell-antitumor activity. TGF-β is a key immunosuppressive cytokine produced by several cell subtypes, including tumor cells, Tregs, stromal cells and myeloid-derived suppressor cells (MDSCs). The pleiotropic activity of TGF-β is of pivotal relevance for the EMT process and cancer progression and, at the same time, is a major suppressor of NKG2D expression, NK cell cytotoxicity, and IFN-γ production of and metastasis in advanced cancer patients [22,81,82,83]. Furthermore, TGF-β-dependent signaling in the tumor microenvironment promotes the conversion of NK cells into type 1 innate lymphoid cell (ILC1) populations, which are unable to control tumor growth and metastasis [84]. In addition, TGF-β and other immunosuppressive molecules produced by tumor cells may recruit and activate immune cells including Tregs [85], MDSCs [86], CD11b+Ly6G+ neutrophils [87], and indoleamine 2,3-dioxygenase 1 (IDO1)-expressing DCs [88] that further suppress NK cell activity. 

Metastatic cells develop specific mechanisms that allow them to survive outside of the tumor nest and to cross the blood to extravasate into distant tissues. Blood is a challenging territory for metastatic cells and there is increasing evidence that the survival of CTCs is greatly enhanced by platelets. Thus, thrombocytopenia inhibits tumor growth and metastasis in mice, whereas depletion of NK cells reverts this anti-metastatic phenotype [89]. Platelets favor the escape of CTCs from NK cells via at least five different mechanisms. They (1) physically shield CTCs from immune recognition [90], (2) secrete immunosuppressive factors including TGF-β [91], (3) transfer MHC class I molecules to cancer cells to establish a state of pseudo-self-tolerance [92], (4) induce the shedding of NKG2D ligands [93], and (5) express NK cell inhibitory ligands such as glucocorticoid-induced TNF-related ligand (GITRL) [94]. Along with the above described immunosuppressive environment, tumor infiltrating lymph nodes and circulating NK cells from patients with metastasis display reduced levels of NK cell activating receptors and, consequently, decreased cytotoxicity and IFN-γ production compared with healthy donors or patients with less-advanced diseases [19,20,95].

To conclude, immune evasion is a major obstacle in the development of effective anticancer therapies. The recent advances in understanding the immune evasion mechanisms developed by disseminated cells are opening new opportunities for novel and more effective cancer immunotherapies.

## 6. Immunotherapy in the Treatment of Metastasis

**Figure 1:**
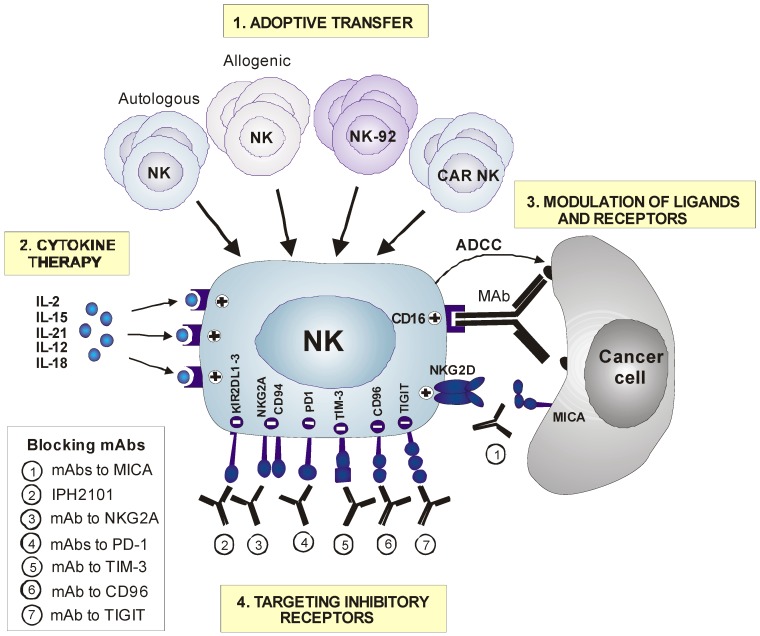
Therapeutic approaches involving Natural Killer (NK) cells to treat metastatic cancer. (**1**) The transfer of expanded and activated NK cells is increasingly used to improve NK cell responses. Autologous NK cells, allogenic NK cells, NK cell lines, such as NK-92 cells, or Chimeric Antigen Receptor (CAR) NK cells may be harnessed as a source of NK cells for adoptive transfer. (**2**) The activity of NK cells may also be stimulated by cytokine or drug treatment. IL-2, IL-15, and IL-21 are the most interesting agents that potentiate NK cell activity. (**3**) Therapeutic approaches that engage NK cell activating receptors are widely used in clinics, particularly, mAbs that engage CD16 receptors and induce Antibody-Dependent Cellular Cytotoxicity (ADCC) activity. Trastuzumab or cetuximab are the most widely employed therapeutic strategies involving NK cells used in clinics. The so-called bispecific and trispecific antibodies may improve ADCC activity by redirecting NK cells and T cells to tumor cells. Targeting other activating NK cell receptors, such as NKG2D, or their ligands, such as MHC class I polypeptide-related sequence A (MICA), are innovative strategies that need to be evaluated in clinical trials. (**4**) Targeting inhibitory receptors and immunosubversive mechanisms developed by cancer cells may release the antitumor potential of NK cells. Blocking antibodies directed against MICA boosts NK cell activity by preventing the shedding of soluble MICA and NKG2D downregulation. Blocking antibodies against inhibitory NK cell receptors or checkpoint proteins, including, but not limited to, Killer cell Immunoglobulin-like Receptors (KIRs) or Natural Killer Group 2A (NKG2A), Programmed Death-1 (PD-1)/PD-L1, TIM-3, CD96, or T cell immunoreceptor with Ig and ITIM domains (TIGIT), have great clinical potential.

The treatment of cancer metastasis is one of the greatest challenges in medicine. Indeed, metastasis is responsible for more than 90% of cancer-associated mortality. Conventional therapeutic strategies, including surgical resection, chemotherapy and radiotherapy are relatively efficient in the elimination of primary tumors, but show limited efficacy in the elimination of metastases. Although immunotherapies are a promising strategy against cancer metastasis, current immunotherapies are mainly focused on T cells, and many toxicity and efficacy complications remain to be solved. NK cells have potent anti-tumor and regulatory activity and are key regulators of T cell-mediated immunity [96], which supports the idea that NK cell immunotherapy may be an alternative or complementary to T cell immunotherapy (Table 1, Figure 1). 

### 6.1. Adoptive Transfer of NK Cells

NK cells are considered ideal targets for adoptive transfer owing to their capability to eliminate tumor cells in a non-MHC and non-tumor antigen-restricted manner. However, the number and the cytotoxic capacity of circulating NK cells is limited and, therefore, it is necessary to expand and activate NK cells before their infusion into patients. In hematopoietic stem cell transplantation, blood NK cells recover very early after transplantation and the number of NK cells correlates with the clinical outcome [97,98]. Initial clinical trials in patients with metastatic cancer showed that the transfer of expanded autologous NK cells in monotherapy was well tolerated, although no clinical response was observed [99,100,101]. Nonetheless, the efficacy of allogeneic NK cells in controlling human cancers is supported by more compelling evidence. The transfer of allogenic NK cells in hematological malignancies, and, particularly, the adoptive transfer of KIR-ligand mismatched donors in patients with Acute Myeloid Leukemia (AML) has shown impressive clinical results [102]. The lack of engagement of inhibitory KIR receptors, by their MHC class I ligands in these patients, showed how the full potential of NK cells may translate to outstanding clinical responses. In solid tumors, initial clinical studies of allogenic NK cell adoptive transfer have shown that this therapy is safe and well-tolerated and may result in a clinical response in metastatic cancer patients [103,104,105,106,107,108]. Nevertheless, the therapeutic adoptive transfer of NK cells in the patient has not yet been definitely developed. There is very limited experience in terms of the adequate conditioning regimen, the source of NK cells, the NK cell activation, and the expansion protocols, or the need to combine therapies to unleash the full anti-tumor potential of NK cells. Thus, many procedures to obtain clinical grade NK cells from peripheral blood, bone marrow, or cord blood have been developed and their quality may vary. In addition, many different approaches to expand and activate NK cells exist. Hence, the priming of NK cells with CTV-1 leukemia cell line lysate CNDO-109 results in enhanced cytotoxicity against NK cell-resistant cell lines and NK cell activation in high-risk patients with AML in a phase I clinical trial [109]. NK cells can also be pre-activated with IL-12, IL-15 and IL-18. This approach has been used to generate the so-called Cytokine-induced memory-like (CIML) NK cells with potent in vitro and in vivo anti-leukemia responses along with improved clinical responses in patients with AML in the context of a first-in-human phase I clinical trial [110]. As indicated in Table 1, a number of different NK-based therapies have been studied or are under consideration. These approaches (and their effectiveness) greatly vary depending on the tumor type, tumor cell phenotype, patient, and feasibility of the treatments. 

CAR T cells are emerging as a new revolution in the treatment of cancer [111]. Similarly, a novel way to boost the therapeutic activity of NK cell transfer is to manipulate and redirect NK cell antitumor activity by chimeric antigen receptor (CAR) engineering. NK cells should be considered an alternative to T cells since they are non-MHC-restricted “serial killers” of tumor cells and, therefore, can be used as universal donors for cancer immunotherapy. Despite the fact that the evaluation of CAR NK cells in solid tumors and metastases are mostly in preclinical studies and initial clinical trials [112,113], CAR NK cells may theoretically show several advantages over CAR T cells [114]. First, CAR NK cells are considered safer than their T cell counterparts. Owing to the fact that NK cells are short-living cells, CAR NK cell therapy is not expected to be associated with long-term problems characteristic of CAR T cell therapy, such as the risk of autoimmunity or malignant transformation. Furthermore, the so-called “cytokine storm” initiated by CAR T cells may be severe, even fatal, and is largely mediated by the production of pro-inflammatory cytokines by T cells. NK cells produce cytokines, including IFN-γ and GM-CSF, with a lower toxicity profile and are considered safer than those produced by T cells. A recent phase I clinical trial evaluating the safety and efficacy of using CAR NK-92 cells targeting CD33 in patients with relapsed and refractory AML showed no major adverse effects, indicating that CAR NK cells may be a safe alternative for CAR T cells [115]. CAR NK cells could be also more efficient in terms of eliminating tumor cells than CAR T cells as they are endowed with spontaneous cytotoxic activity provided by an array of activating receptors that may target cancer cells in an MHC and antigen-unrestricted manner, which may potentiate the activity of the CAR. Additionally, NK cells are endowed with intrinsic ADCC activity that enables the use of combination therapies involving CAR NK cells and monoclonal antibodies. Nevertheless, there are some drawbacks to the use of CAR NK cells [114]. NK cells are difficult to obtain, expand and manipulate as they show quite low transfection efficiency even when viral vectors are used [116]. 

NK cell lines, such NK-92 cells, may be an alternative source of NK cells for cancer immunotherapy. They have several advantages over autologous or allogenic NK cells. NK-92 cells are an unlimited, homogeneous and well-defined population of highly active NK cells that can be obtained at a low cost [117]. However, NK-92 cells have several disadvantages, such as their lack of typical NK cell activating receptors, including CD16, NKp44, and NKp46, and, as with any other tumor cell line, NK-92 cells carry multiple cytogenetic abnormalities and are latently infected by Epstein-Barr virus [118]. Hence, NK cell lines have to be irradiated before infusion for safety. Unmodified NK-92 cells have been employed to treat patients with advanced cancers, with encouraging responses observed in patients with advanced lung cancer [119]. Furthermore, NK-92 cells may constitute a suitable source of NK cells for CAR engineering since they show a transfection efficiency of about 50% even with non-viral methods. Preclinical data demonstrate that CAR NK-92 cells targeting the receptor tyrosine kinase ErbB2/HER2 or the epidermal growth factor receptor (EGFR) protect mice against glioblastoma [120,121,122]. In a preclinical study, CAR NK-92 cells targeting EGFR combined with oncolytic herpes simple virus 1 have been shown to be a novel strategy for breast cancer brain metastases [123]. CAR NK-92 cells targeting ERbB2/HER2 also reduced lung metastasis in a renal carcinoma murine model, showing their potential in relation to the treatment of the disseminated disease [113]. 

### 6.2. Cytokine-Based NK Cell Therapy

IL-2 was one of the first cytokines employed to treat human cancers. It showed great therapeutic potential in controlling metastatic diseases owing to its pleiotropic capability of boosting T and NK cell antitumor activity [124]. Although high doses of IL-2 improved the survival of a fraction of treated patients with metastatic renal cancer and melanomas [125], it caused life-threatening toxicities including vascular leak syndrome (VLS) [124]. Furthermore, both high- and low-dose IL-2 therapy induced the expansion in patients of immunosuppressive CD4+CD25+Foxp3+ Treg cells expressing the high affinity IL-2 receptor α-chain (CD25) [126,127,128]. Thus, IL-2 monotherapy is not the optimal or standard treatment for these patients and efforts have been made to further improve the efficacy of IL-2 therapy in combination with other anticancer immunotherapy regimens [124]. Early combination therapies were initiated incorporating immune cells such as lymphokine activated killer (LAK) cells and T cells. Co-administration of LAK cells with IL-2 yielded a clinical response rate of 20 to 35%, however, mostly with a transient response in solid tumors [129]. Subsequently, IL-2 was also used in conjunction with adoptive T cell therapy, significantly improving efficacy [130,131,132]. Similarly, IL-2 is also frequently associated with the adoptive transfer of NK cells for the promotion of the expansion of NK cells in vivo. 

Cytokines that activate NK cell development, survival and activity without activating Tregs (such as IL-12, IL-15, IL-18, and IL-21) are now being studied [133]. A compelling body of studies supports the idea that IL-15 is a cytokine with a high potential to be harnessed in cancer therapy [134]. First, while IL-15 shared the strong stimulatory effect on cytotoxic immune cells with IL-2, treatment with IL-15 did not result in immunosuppression and did not cause vascular leak syndrome in mice or nonhuman primates [135,136]. Second, preclinical studies demonstrated that IL-15 induced prolonged expansion and activation of NK cells and CD8 memory T cells, therefore resulting in tumor regression, decreased metastasis, and increased survival in several experimental models [135,136,137,138,139,140]. These observations supported the use of IL-15 as an alternative treatment in patients with metastatic malignancies and led to the implementation of IL-15-based therapies in several clinical trials (source https://clinicaltrials.gov/). These involved the administration of IL-15 alone or in combination with NK transfer or chemotherapy in patients with solid tumors and hematological malignancies [134]. A hyper-proliferation and an increased number of circulating NK cells was reported in the first in-human phase I clinical trial of IL-15 in patients with metastatic malignant melanoma and metastatic renal cell cancer [141]. Preliminary antitumor evaluation showed no objective responses, but the clearance of lung metastases in two patients was observed [141]. In a phase I/II clinical trial, IL-15-stimulated NK cells induced a clinical response in four out of six pediatric patients with solid refractory tumors [142]. Despite these promising clinical results, therapies involving IL-15 must deal with short in vivo half-life of the cytokine. Several approaches have been developed to overcome this limitation, largely by generating super agonist IL-15/IL-15Rα conjugates that exhibit greater activity than that of naïve IL-15 [134]. 

Other cytokines that activate NK cells without stimulating Tregs are being evaluated. IL-12 and IL-18 showed, in monotherapy, rather limited anti-tumor activity in humans, while a combination of IL-12/IL-18/IL-15 induced sustained in vitro and in vivo effector functions in human and mice NK cells respectively [143]. IL-21 represents another interesting cytokine for cancer immunotherapy as it induces the antitumor activities of CD8 T cells, NK cells, and NKT cells [144,145]. It showed antitumor activity in several preclinical cancer models [146,147,148,149] and in a phase II study in patients with metastatic melanomas [150]. However, at high concentrations, IL-21 can also lead to dose-limiting side effects including 3/4 grade granulocytopenia and liver toxicity. It may also perform pro-inflammatory activities in many tissues and promote colitis-associated colon cancer [151]. Thus, a more profound understanding of the biology of IL-21 is needed to ensure its maximal therapeutic benefit.

Multiple experimental and preclinical studies reporting the effectiveness of several drugs in the boosting of NK cell antitumor activity have been published, including that of inhibitors of TGF-β, antagonists of the high adenosine receptor A_2_, immunomodulatory drugs such as lenalidomide, DNA complexes (CpG), demethylating agents, histone deacetylases inhibitors, etc. [12]. Despite such efforts, the clinical potential of these NK cell-activating strategies in the treatment of metastasis remains to be established. 

### 6.3. Modulation of NK Cell Receptors or Ligands

Activating and inhibitory receptors or their ligands can be targeted by monoclonal antibodies (mAbs) to increase therapeutic NK cell activity. The most widely used strategy in cancer immunotherapy is to employ tumor-specific antibodies that promote ADCC through the ligation of CD16 receptors on NK cells. Rituximab (mAb to the B cell marker CD20), trastuzumab (mAb to ErbB2/HER2) and cetuximab (mAb to EGFR) have demonstrated marked efficacy in the treatment of various solid and hematological tumors [8]. No therapeutic effect was achieved in mice deficient in Fc receptors [152] and polymorphisms in the genes encoding these receptors modulated their clinical response [153,154], suggesting that Fc receptors and ADCC play a crucial role in the therapeutic activity of these mAbs. 

Trastuzumab, the first antibody developed against a growth factor, was successfully introduced in the treatment of HER2+ breast cancers, which represent 15–20% of this tumor type. Trastuzumab combined with chemotherapy has dramatically improved the survival of HER2+ patients [155] and even trastuzumab in monotherapy improves the clinical response and survival of patients with metastatic breast cancer [156]. However, high proportions of patients relapse and experience brain metastasis after treatment with trastuzumab. Currently, several combinations of strategies for improving the efficacy of mAbs are being tested. For example, several clinical trials are now assessing the safety and efficacy of the combination of autologous NK cells with trastuzumab in patients with HER2+ breast or gastric cancers (source https://clinicaltrials.gov/). 

A step toward improving ADCC activity for cancer immunotherapy is the development of the so-called bispecific or trispecific antibodies. Bispecific antibodies can bind simultaneously two different antigens, which leads to a wide range of applications including redirecting T cells or NK cells to tumor cells. The so-called bispecific trifunctional antibodies have dual specificity while preserving ADCC potential. The most successful example of this class of new drugs is catuxomab, which consists of one “Fab” recognizing the epithelial cell adhesion molecule (EpCAM), the other "Fab" recognizing a CD3 antibody, and the Fc region binding to NK cells or macrophages. Catuxomab is now approved in the European Union for the treatment of patients with malignant ascites from EPCAM-positive tumors [157].

Targeting NKG2D ligands may also stimulate NK cell activity. Chronic exposure to MICA/B downregulates NKG2D expression at the surface of NK and CD8 T cells, suppressing their activity. The MICA/B targeting antibody IPH4301 mediates potent NK cell-stimulatory effects in vitro and in murine models and prevents NKG2D downregulation [12]. Recently, antibodies targeting the α3 domain of MICA, preventing soluble MICA/B shedding, have been shown to inhibit tumor growth in multiple murine models and to reduce human melanoma metastases in a humanized mouse model, mainly by NK cell activation through NKG2D and CD16 receptors [158]. These antibodies are good candidates for clinical testing.

### 6.4. Targeting Inhibitory Receptors

Blocking the inhibitory pathways dampening NK cell function in cancer is an encouraging strategy for NK cell immunotherapy [159] (Figure 1). Checkpoint inhibitors are currently the most promising drugs for cancer immunotherapy. Treatment with anti-CTLA4 and PD1/PD-L1 antibodies showed impressive results in patients with metastatic cancer, causing durable tumor regression in a significant group of patients [160]. A combination of both checkpoint inhibitors had cooperative clinical effects [161]. The clinical benefit of these antibodies is thought to mainly rely on the reactivation of exhausted T cells. However, NK cells also express checkpoint molecules and, consequently, they may also contribute to the observed therapeutic benefit. In fact, the PD1/PDL1 blockade in several mouse models elicited a strong NK cell-mediated response that was indispensable for the full therapeutic effect of immunotherapy [162]. Further, NK cells positively regulated the abundance of the stimulatory DCs through the production of cytokines such as FLT3LG, CCL5 and XCL1 in mouse and human tumors. Both infiltrating NK cells and stimulatory DCs correlated with increased overall survival and responsiveness to anti-PD1 immunotherapy in melanoma patients [80,163]. PD1 was also shown to inhibit NK cell activity in multiple myeloma and digestive cancers, particularly in patients with advanced diseases and PD1 blockade restored NK cell cytotoxicity and IFN-γ production [78,79]. These recent data suggest that NK cells may contribute to checkpoint inhibitor clinical efficacy, particularly in hematological malignancies and patients with metastases. This matter clearly warrants further investigation. 

Overexpression of MHC class I molecules in cancer cells inhibits NK cell activation through the interaction with KIR receptors and blocking this interaction may have therapeutic benefits for cancer patients. IPH2101 is an antibody that binds to the high affinity inhibitory receptors KIR2DL-1, KIR2DL-2, and KIR2DL-3, blocking their interaction with HLA-C group 1 and 2 allotypes, which boosts NK cell anti-tumor activity in preclinical studies [164]. Nevertheless, IPH2101 failed to demonstrate any clinical benefit in patients with smoldering multiple myeloma in a clinical trial, probably due to an unexpected NK cell inhibitory effect [165]. Monalizumab, an antibody that blocks NKG2A-HLA-E interaction, has been shown to induce NK cell antitumor activity against leukemia cells from patients with chronic lymphocytic leukemia [166] and is currently being tested for efficacy and efficiency in several hematological malignancies and advanced solid tumors (source: https://clinicaltrials.gov).

The inhibitory receptors TIGIT and CD96 (TACTILE) have recently emerged as new checkpoints for cancer immunotherapy. They inhibit NK cell activity and counterbalance CD226 activation of NK cells. TIGIT was associated with NK cell exhaustion in tumor bearing mice and patients with colon cancer and TIGIT blockade promoted NK cell anti-tumor immunity in several mouse models [167]. CD96 blockade also inhibited experimental metastases in three different murine models, the therapeutic effect of which depended on NK cells, DNAM-1, and IFN-γ, but was independent of Fc receptors [168]. Furthermore, blocking CD96 in Tigit^−/−^ mice significantly reduced experimental and spontaneous metastases. Finally, the TIM-3 expression on NK cells was upregulated on metastases and correlated with a poor prognosis in patients with lung adenocarcinoma, melanomas, and gastric cancer; and TIM-3 blockade restored NK cell anti-tumor activity ex vivo [169,170,171]. 

To conclude, a more profound understanding of the evasion mechanisms developed by tumor cells is needed in order to develop new strategies for cancer immunotherapy. Due to the wide variety of immune evasion mechanisms specifically developed by each type of cancer, it is likely that the abovementioned, novel NK cell activating therapies may only benefit a specific group of patients and a more personalized therapeutic approach might be required in the future.

## 7. Conclusions

Cancer metastasis is the leading cause of cancer-related death. Current cancer therapies, including surgery, chemotherapy and radiotherapy, have shown a limited capacity for improving the survival of metastatic patients. Conversely, T cell-mediated immunotherapy has obtained impressive clinical results in some metastatic patients, particularly melanoma patients. Further studies aimed at overcoming the immunosuppressive tumor microenvironment and managing the adverse effects induced by possible self-reactive T cells are needed to fully exploit the therapeutic potential of these cells. As an alternative, or even as a complement, the use of NK cell immunotherapy shows great potential in the treatment of metastatic patients. NK cells harbor innate and potent anti-metastatic activity, which is independent of MHC restriction and tumor antigen expression, and they may be envisioned as a universal source of antitumor cells for cancer immunotherapy. Now, the challenge is to translate the increasing amount of basic research into new therapeutic strategies. Initial NK cell immunotherapies for metastatic patients have shown encouraging clinical results and some NK cell immunotherapies could be associated with a better toxic profile than that of T cells. Nevertheless, further experimental and clinical studies have to be performed in order to completely understand the antitumor and anti-metastatic properties of NK cells and the most effective way to fully unleash their therapeutic potential.

## Figures and Tables

**Table 1:** The most innovative NK cell immunotherapies for targeting metastasis.

Therapy	Advantages	Disadvantages	References
Adoptive transfer			
Autologous NK cells	Universal use. Safe	Low efficacy	[97,98,99,100,101]
Allogenic NK cells	Highly effective against some KIR-ligand mismatch malignancies	In clinical evaluation for metastatic cancer; no standard protocols or products	[102,103,104,105,106,107,108,109,110]
CAR NK cells	Highly potentiate NK cell antitumor activity; likely to be more efficient and safer than CAR T cells	Difficult to manipulate and expand In clinical evaluation for metastatic cancer	[111,112,113,114,115,116]
NK cell lines	Unlimited, homogeneous, well-defined, and highly active population of NK cells; low cost	Low efficacy; safety concerns; need to be irradiated	[117,118,119,120,121,122,123]
Cytokine-based therapy			
IL-2	Boost NK cell and T cell activity; significant efficacy in a proportion of melanoma and renal metastatic patients	Toxic at high doses; activation of Tregs	[124,125,126,127,128,129,130,131,132]
IL-15	Similar antitumor activity to IL-2 without activating Tregs and with better toxic profile	In clinical evaluation for metastatic cancer	[133,134,135,136,137,138,139,140,141,142,143]
IL-21	Boost NK cell and T cell activity without activating Tregs, potential combination with MAbs	Low knowledge of IL-21 biology; possible unexpected effects	[144,145,146,147,148,149,150,151]
Modulation of receptors or ligands			
Tumor-targeting MAbs	Redirect NK cell activity against specific tumors Promote ADCC; improve survival of metastatic patients	Needs combination to improve efficacy	[152,153,154,155,156]
Bispecific, trispecific Abs	Multiple targets; redirect NK cells and T cells against specific tumors; improve anti-tumor capacity	Possible off-target effects	[157]
mAb to NKG2D (IPH4301)	Prevent immune evasion	Lack of clinical experience	
mAb to MICA	Prevent soluble MICA shedding and NKG2D downregulation	Lack of clinical experience	[158]
Targeting inhibitory receptors			
PD1/PD-L1 blockade	Impressive clinical results in some metastatic patients; manageable toxicity	Lack of knowledge of the role of NK cells and the clinical benefits	[159,160,161,162,163]
mAbs to KIRs	Safe; boost NK cell activity against tumor cells	Low efficacy; need to be combined	[164,165]
mAbs to NKG2A (monalizumab)	Boost NK cell activity against tumor cells	In clinical evaluation	[166]
TIGIT/CD96 blockade	Boost NK cell activity against tumor cells and metastasis	In preclinical studies	[167,168]
TIM-3 blockade	Boosts NK cell activity against tumor cells and metastasis	In preclinical studies	[169,170,171]

NK: Natural Killer, CAR: Chimeric Antigen Receptor, IL: Interleukin, ADCC: Antibody-Dependent Cellular Cytotoxicity, NKG2D: Natural killer group 2D, MICA: MHC class I polypeptide-related sequence A, KIR: Killer cell Immunoglobulin-like Receptor, TIGIT: T cell immunoreceptor with Ig and ITIM domains, PD1: Programmed Death 1, TIM-3: T-cell Immunoglobulin and Mucin-domain containing-3.
